# Fluorescent sensor for water based on photo-induced electron transfer and Förster resonance energy transfer: anthracene-(aminomethyl)phenylboronic acid ester-BODIPY structure†

**DOI:** 10.1039/c9ra02686j

**Published:** 2019-05-16

**Authors:** Daisuke Jinbo, Keiichi Imato, Yousuke Ooyama

**Affiliations:** Department of Applied Chemistry, Graduate School of Engineering, Hiroshima University Higashi-Hiroshima 739-8527 Japan yooyama@hiroshima-u.ac.jp +81 824 24 5494 +81 824 24 7689

## Abstract

An anthracene-(aminomethyl)phenylboronic acid ester-BODIPY (DJ-1) was designed and developed as a fluorescent sensor based on photo-induced electron transfer (PET) and Förster resonance energy transfer (FRET) for the detection of a trace amount of water in solvents, where the anthracene skeleton and BODIPY skeleton are the donor fluorophore and the acceptor fluorophore in the FRET process, respectively. It was found that the addition of water to organic solvents containing DJ-1 causes both the suppression of PET in the anthracene-(aminomethyl)phenylboronic acid ester as the PET-type fluorescent sensor skeleton and the energy transfer from the anthracene skeleton to the BODIPY skeleton through a FRET process, thus resulting in the enhancement of the fluorescence band originating from the BODIPY skeleton. This work demonstrates that the PET/FRET-based fluorescent dye composed of the donor fluorophore possessing PET characteristics and the acceptor fluorophore in the FRET process can act as a fluorescent sensor with a large SS for the detection of a trace amount of water in solvents.

## Abstract 2

An anthracene-(aminomethyl)phenylboronic acid ester-BODIPY (DJ-1) structure was developed as a fluorescent sensor based on photo-induced electron transfer (PET) and Förster resonance energy transfer (FRET) for detection of water in solvents.

## Graphical abstract

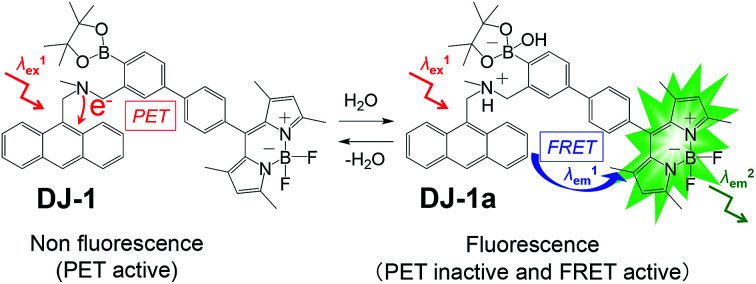

## Introduction

**Fig. 1:**
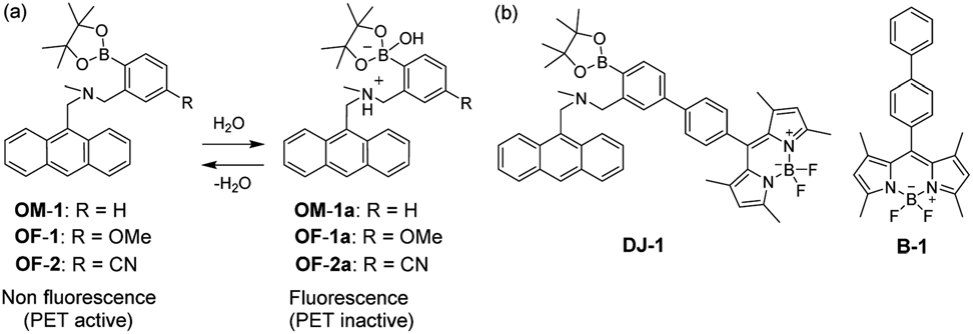
(a) Proposed mechanisms of PET-type fluorescent sensor OM-1, OF-1, and OF-2 for the detection of water in solvent. (b) Chemical structures of anthracene-(aminomethyl)phenylboronic acid ester-BODIPY DJ-1 possessing PET and FRET characteristics, and BODIPY B-1.

Development of fluorescent sensors for the visualization as well as detection and quantification of water in samples and products, such as solutions, solids, and gas or water on the surface of a substrate, has been of considerable concern in recent years for not only fundamental study in analytical chemistry, photochemistry, and photophysics, but also their potential applications to environmental and quality control monitoring systems and industry.^1^ To date, some kinds of fluorescent sensor for water based on ICT (intramolecular charge transfer),^2,3^ PET (photo-induced electron transfer),^4,5^ or ESIP (excited state intramolecular proton transfer)^6^ have been designed and synthesized, and the optical sensing properties of these fluorescent sensors for the detection and quantification of water have been investigated from the viewpoints of the relationship between ICT, PET, or ESIP characteristics and the intermolecular interaction of the sensor with water molecules. Among them, in particular, the PET-type fluorescent sensor based on the fluorescence enhancement system is useful for the detection and quantification of a trace amount of water in organic solvents because the fluorescence intensity of the sensor increases as a function of water content in organic solvents, which is attributed to the suppression of PET from the electron donor part to the photo-excited fluorophore due to the intermolecular interaction between the fluorescent sensors and water molecules. Actually, in our previous work, anthracene-(aminomethyl)phenylboronic acid pinacol esters (OM-1, OF-1 and OF-2) was designed and synthesized as PET-type fluorescent sensors for a trace amount of water (Fig. 1a).^5*a*^ The PET takes place from the nitrogen atom of amino moiety to the photoexcited fluorophore skeleton in the absence of water, leading to the fluorescence quenching. The addition of water to organic solvents containing PET-type fluorescent sensor causes a drastic enhancement of fluorescence, which is attributed to the suppression of PET. The nitrogen atom of amino moiety is protonated or strongly interacts with water molecule, leading to the formation of PET inactive species such as OM-1a. Thus, the fluorescence enhancement system based on the PET method is useful for the detection and quantification of a trace amount of water in organic solvents. However, the PET-type fluorescent sensor such as OM-1 usually has the disadvantage of very small Stokes shift (SS), leading to serious self-quenching and fluorescence detection errors due to photoexcitation and scattering lights from the excitation source; SS is the difference in wavelength or frequency units between the maximum of the first photoabsorption band and the maximum of fluorescence band. On the other hand, Förster resonance energy transfer (FRET)-type sensor is useful for applications in biochemistry and environmental research such as nucleic acid and ion analysis, signal transduction, and light harvesting, as well as for designing ratiometric fluorescent sensors.^7,8^ FRET is well described as an energy transfer process between an excited-state donor fluorophore and a ground-state acceptor fluorophore linked together by a non-conjugated spacer, and as the result, the emission spectrum from acceptor fluorophore is observed. For an effective FRET, a strong spectral overlap between the donor emission and the acceptor absorption is required. Consequently, the pseudo-SS between the maximum of donor absorption band and the maximum of acceptor fluorescence band of FRET-type sensors is larger than the SS of either the donor or acceptor fluorophores, leading to an effective avoidance of the self-quenching and fluorescence detection errors due to photoexcitation and scattering lights from the excitation source. However, to the best of our knowledge there are no reports for the PET/FRET-based fluorescent sensor composed of the donor fluorophore possessing PET characteristics and the acceptor fluorophore in FRET process. It is expected that the development of PET/FRET-based fluorescent sensor can allow creation of fluorescent sensor system with large SS for the detection of a trace amount of water in solvents, that is, the PET/FRET-based fluorescent sensor has the advantage over PET-type fluorescent sensor^4,5^ which usually has the disadvantage of very small SS.

Thus, in this work, in order to develop a fluorescent sensor possessing large SS for a trace amount of water in solvents, we have designed and synthesized an anthracene-(aminomethyl)phenylboronic acid ester-BODIPY (DJ-1) as a fluorescent sensor based on PET and FRET characteristics, where the anthracene skeleton and BODIPY skeleton are the donor fluorophore and the acceptor fluorophore in the FRET process, respectively (Fig. 1b). That is, it is expected that the addition of water to organic solvents containing DJ-1 causes both the suppression of PET in the anthracene-(aminomethyl)phenylboronic acid ester as the PET-type fluorescent sensor skeleton and the energy transfer from anthracene skeleton to BODIPY skeleton through a FRET process, and thus resulting in the enhancement of the fluorescence band originating from BODIPY skeleton. Herein, we report the optical sensing property of DJ-1 for the detection of water in solvents based on the PET and FRET characteristics of the anthracene-(aminomethyl)phenylboronic acid ester-BODIPY.

## Results and discussion

**Scheme 1:**
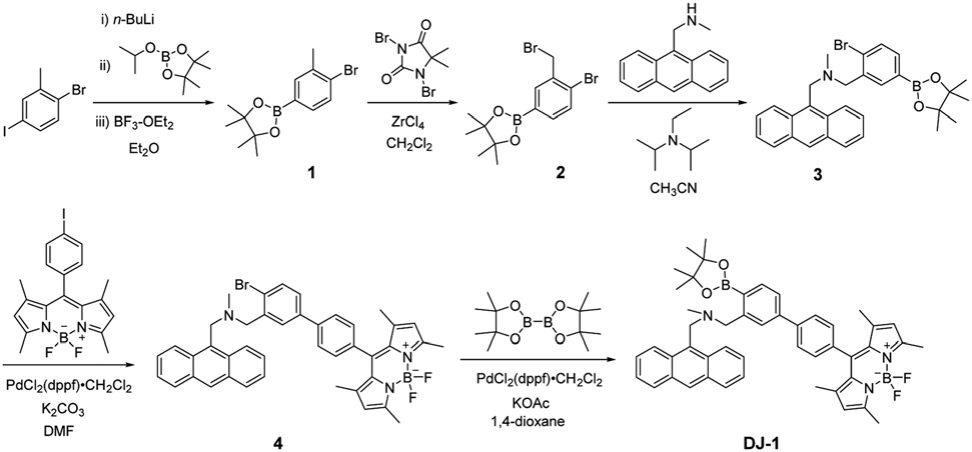
Synthesis of DJ-1.

The PET/FRET-type fluorescent sensor DJ-1 was synthesized according to a stepwise synthetic protocol (Scheme 1). Compound 1 was prepared through treatment with *n*-BuLi and then 2-isopropoxy-4,4,5,5-tetramethyl-1,3,2-dioxaborolane followed by treatment with BF_3_–OEt_2_. ZrCl_4_-catalyzed benzylic bromination^9^ of 1 with 1,3-dibromo-5,5-dimethylhydantoin gave compound 2. The reaction of 9-(methylaminomethyl)anthracene with 2 in the presence of *N*,*N*-diisopropylethylamine yielded compound 3. Compound 4 was prepared by Suzuki coupling of BODIPY derivative having iodophenyl substituent with compound 3. Finally, we obtained DJ-1 from 4 with bis(pinacolato)diboron *via* the Miyaura boronation reaction. The BODIPY B1 as reference was prepared by Suzuki coupling of the BODIPY derivative having iodophenyl substituent with phenylboronic acid according to literature^10^ (Fig. 1, see Scheme S1 for the synthesis, ESI†).

**Fig. 2:**
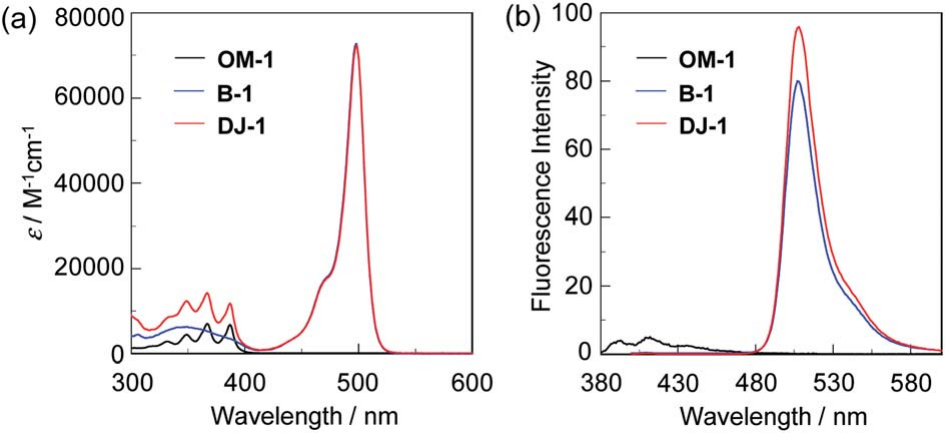
(a) Photoabsorption and (b) fluorescence (*λ*^ex^ = 366 nm for OM-1 and 367 nm for B-1 and DJ-1, respectively) spectra of OM-1 (*c* = 2.0 × 10^−5^ M), B-1 (*c* = 4.0 × 10^−6^ M), and DJ-1 (*c* = 4.0 × 10^−6^ M) in acetonitrile.

The photoabsorption and fluorescence spectra of OM-1, B-1 and DJ-1 in acetonitrile are shown in Fig. 2. OM-1 shows a photoabsorption band in the ranges of 300 nm to 400 nm originating from the anthracene skeleton, and B-1 shows a photoabsorption band in the ranges of 420 nm to 520 nm originating from the BODIPY skeleton. In addition, for B-1 a feeble and broad photoabsorption band was observed in the ranges of 300 nm to 400 nm. The molar extinction coefficient (*ε*_max_) for the photoabsorption maximum (*λ*^abs^_max_ = 498 nm) of B-1 is 72 600 M^−1^ cm^−1^, which is significantly higher than that (*λ*^abs^_max_ = 366 nm, *ε*_max_ = 6800 M^−1^ cm^−1^) of OM-1. On the other hand, DJ-1 shows two photoabsorption bands in the ranges of 300 nm to 400 nm (*λ*^abs^_max_ = 367 nm, *ε*_max_ = 14 200 M^−1^ cm^−1^) and 420 nm to 520 nm (*λ*^abs^_max_ = 498 nm, *ε*_max_ = 72 200 M^−1^ cm^−1^), which are assigned to the anthracene skeleton and the BODIPY skeleton, respectively (Fig. 2a). For the corresponding fluorescence spectra, OM-1 and B-1 exhibit a fluorescence maximum (*λ*^fl^_max_) at 412 nm and 507 nm, by the photoexcitation (*λ*^ex^) at 366 nm and 367 nm, respectively (Fig. 2b). It is worth mentioning here that the edge for the fluorescence band of OM-1 reached 500 nm, that is, the photoabsorption spectrum originating from the BODIPY skeleton of B-1 has spectral overlap with the fluorescence spectrum originating from the anthracene skeleton of OM-1. The fact suggests that for DJ-1 the FRET from the anthracene skeleton as the donor fluorophore to the BODIPY skeleton as the acceptor fluorophore occurs by the photoexcitation of the anthracene skeleton. In fact, DJ-1 exhibits an only fluorescence band with the *λ*^fl^_max_ at 508 nm in the ranges of 480 nm to 600 nm originating from the BODIPY skeleton by the photoexcitation (*λ*^ex^ = 367 nm) of the anthracene skeleton, as well as the photoexcitation (*λ*^ex^ = 472 nm) of the BODIPY skeleton (Fig. S7, ESI†). In addition, the pseudo-SS value of DJ-1 between the *λ*^abs^_max_ of the anthracene skeleton and the *λ*^fl^_max_ of the BODIPY skeleton is 7563 cm^−1^ (141 nm), which is significantly higher than that (395 cm^−1^) of OM-1 and that (356 cm^−1^) of B-1. Therefore, it is expected that the addition of water to organic solvents containing DJ-1 causes both the suppression of PET in the anthracene-(aminomethyl)phenylboronic acid ester and the energy transfer from anthracene skeleton to BODIPY skeleton through a FRET process, and thus resulting in the enhancement of the fluorescence band originating from BODIPY skeleton.

**Fig. 3:**
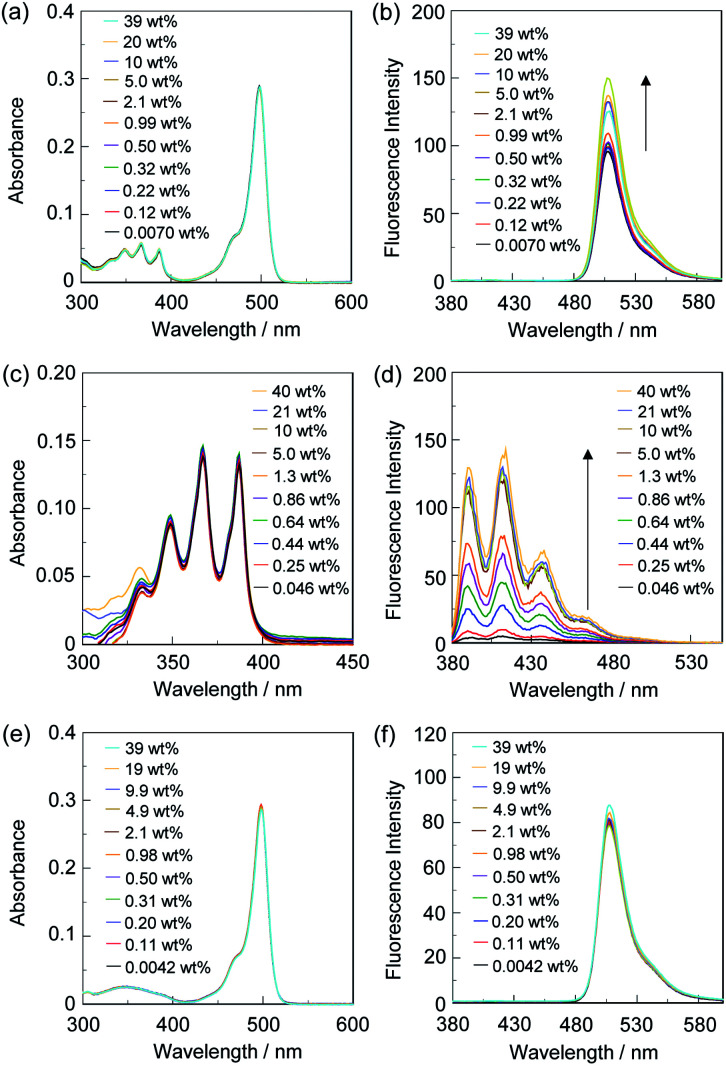
(a) Photoabsorption and (b) fluorescence spectra (*λ*^ex^ = 367 nm) of DJ-1 (*c* = 4.0 × 10^−6^ M) in acetonitrile containing water (0.0070–39 wt%). (c) Photoabsorption and (d) fluorescence spectra (*λ*^ex^ = 366 nm) of OM-1 (*c* = 2.0 × 10^−5^ M) in acetonitrile containing water (0.046–40 wt%). (e) Photoabsorption and (f) fluorescence spectra (*λ*^ex^ = 367 nm) of B-1 (*c* = 4.0 × 10^−6^ M) in acetonitrile containing water (0.0042–39 wt%).

**Fig. 4:**
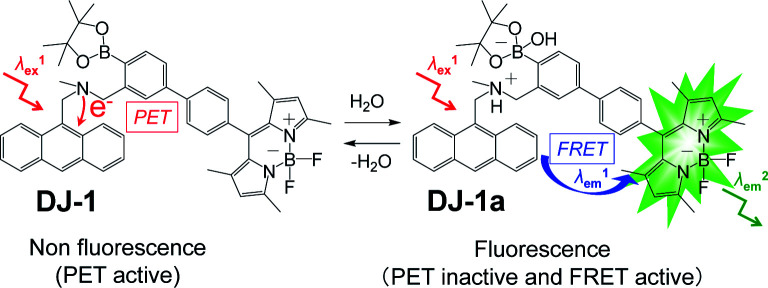
Proposed mechanisms of PET/FRET-type fluorescent sensor DJ-1 for the detection of water in solvent.

Thus, in order to investigate the optical sensing ability of DJ-1 for water in a solvent, the photoabsorption and fluorescence spectra of OM-1 and B-1 as well as DJ-1 were measured in acetonitrile that contained various concentrations of water (Fig. 3). The photoabsorption spectra of DJ-1 show unnoticeable changes upon addition of water to the acetonitrile solution (Fig. 3a). On the other hand, DJ-1 exhibits an enhancement of fluorescence band at 508 nm originating from the BODIPY skeleton by the photoexcitation (*λ*^ex^ = 367 nm) of the anthracene skeleton upon addition of water to the acetonitrile solution (Fig. 3b). The enhancement of the fluorescence band levels off when the water content becomes 5.0 wt%. This result indicates that the enhancement of fluorescence band originating from the BODIPY skeleton is attributed to both the suppression of PET in the anthracene-(aminomethyl)phenylboronic acid ester and the occurrence of FRET from the excited-state anthracene fluorophore to the ground-state BODIPY fluorophore upon addition of water to the acetonitrile solution. As more evidence for the FRET process in DJ-1, the fluorescence spectra of DJ-1 by the photoexcitation (*λ*^ex^ = 472 nm) of the BODIPY skeleton did not undergo appreciable changes in intensity and shape of the fluorescence band originating from BODIPY skeleton upon addition of water to the acetonitrile solution (Fig. S7, ESI†). On the other hand, the photoabsorption spectra of OM-1 did not undergo appreciable changes upon addition of water to the acetonitrile solution as with the case of DJ-1 (Fig. 3c), but the fluorescence spectra of OM-1 underwent an increase in intensity at around 415 nm with the increase in the water content, which is attributed to the fluorescence emission originating from the anthracene skeleton due to the suppression of PET (Fig. 3d). Moreover, it is worth mentioning here that the photoabsorption and fluorescence spectra of B-1 did not undergo appreciable changes upon the addition of water to the acetonitrile solution (Fig. 3e, f, and S8, ESI†). Consequently, as shown in Fig, 4, these facts strongly indicate that for DJ-1 the enhancement of the fluorescence band upon addition of water to the solvent is due to both the suppression of PET in the donor fluorophore (anthracene-(aminomethyl)phenylboronic acid ester) and the occurrence of FRET from the excited-state donor fluorophore and the ground-state acceptor fluorophore (BODIPY skeleton), that is, DJ-1 can act as a fluorescent sensor for water based on PET and FRET characteristics (Fig. 4).

**Fig. 5:**
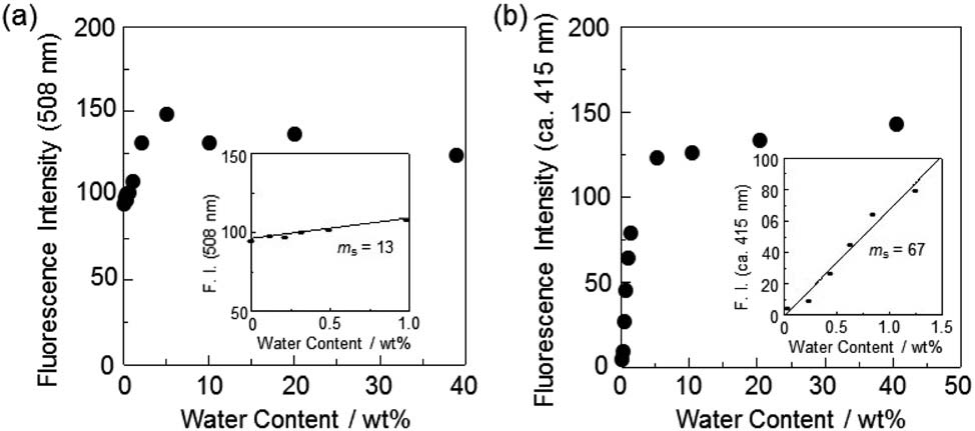
Fluorescence peak intensity at (a) 508 nm of DJ-1 (*λ*^ex^ = 367 nm) and (b) around 415 nm of OM-1 (*λ*^ex^ = 366 nm) as a function of water content below 40 wt% in acetonitrile. Inset in (a): fluorescence peak intensity at 508 nm of DJ-1 (*λ*^ex^ = 367 nm) as a function of water content below 1.0 wt% in acetonitrile. Inset in (b): fluorescence peak intensity at around 415 nm of OM-1 (*λ*^ex^ = 366 nm) as a function of water content below 1.3 wt% in acetonitrile.

**Fig. 6:**
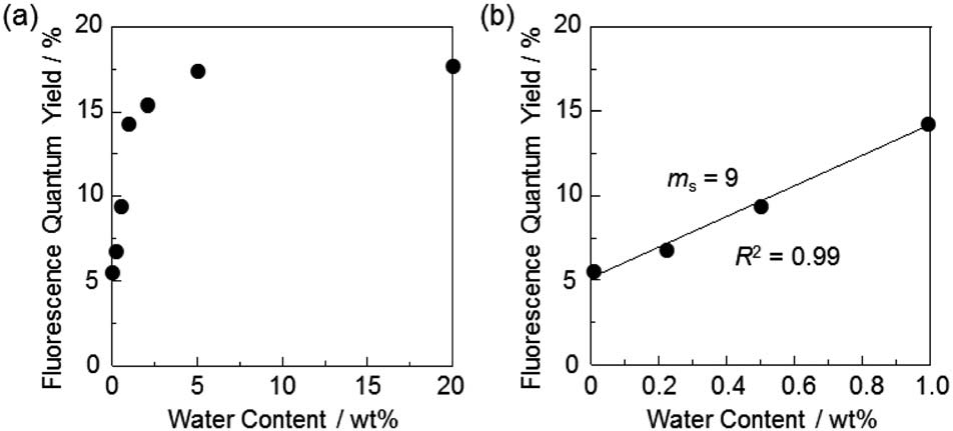
Fluorescence quantum yield of DJ-1 by photoexcitation at 367 nm as a function of water content (a) below 20 wt% and (b) 1.0 wt% in acetonitrile.

In order to estimate the sensitivity and accuracy of DJ-1 as a PET/FRET-type fluorescent sensor for the detection of water in solvent, the changes in fluorescence intensity were plotted against the water fraction in acetonitrile (Fig. 5a). The plot for the low water content region below 1.0 wt% demonstrated that the fluorescence peak intensity at 508 nm increased linearly as a function of the water content (Fig. 5a inset). Indeed, the correlation coefficient (*R*^2^) value for the calibration curve is 0.96, which indicates the good linearity. The enhancement of the fluorescence peak intensity levels off in the water content region greater than 5.0 wt%, which is similar to the case of OM-1 (Fig. 5b). In addition, we performed the measurement of fluorescence quantum yield (*Φ*_fl_) for DJ-1 in the acetonitrile solution with various water content. Indeed, these *Φ*_fl_ values are in good agreement with the intensity of the fluorescence spectra (Fig. 6). These facts also indicate that the fluorescence sensing mechanism of DJ-1 for water is based on the suppression of PET and occurrence of FRET by water molecules. Thus, the detection limit (DL) was determined from the plot of the fluorescence intensity at 508 nm *versus* the water fraction in the low water content region below 1.0 wt% (DL = 3.3*σ*/*m*_s_, where *σ* is the standard deviation of the blank sample and *m*_s_ is the slope of the calibration curve). The *m*_s_ and DL values of DJ-1 are 13 and 0.25 wt%, which are inferior to those (*m*_s_ = 67, DL = 0.04 wt%) of the PET-type fluorescent sensor OM-1 (Fig. 5b).^5*a*^ The *m*_s_ and DL values of PET/FRET-type fluorescent sensor DJ-1 may be dependent on the non-conjugated spacer between the donor fluorophore and the acceptor fluorophore, that is, the substituent on the phenylboronic acid pinacol ester. In fact, the *m*_s_ value (55) and DL value (0.06 wt%) of OF-1 having a methoxy group as an electron-donating substituent is inferior to that of OM-1, but the *m*_s_ value (382) and DL value (0.009 wt%) of OF-2 having a cyano group as an electron-withdrawing substituent is superior to those of OM-1 and OF-1.^5*d*^ Therefore, it is expected that the *m*_s_ value and DL values of a PET/FRET-type fluorescent sensor for the detection of water can be improved by modifying the non-conjugated spacer between the donor fluorophore and the acceptor fluorophore.

## Conclusions

We have designed and developed an anthracene-(aminomethyl)phenylboronic acid ester-BODIPY (DJ-1) as a fluorescent sensor possessing large SS based on PET and FRET characteristics for the detection of a trace amount of water in solvents. It was found that the enhancement of fluorescence band originating from the BODIPY skeleton upon addition of water to the acetonitrile solution is due to both the suppression of PET in the donor fluorophore (anthracene-(aminomethyl)phenylboronic acid ester) and the occurrence of FRET from the excited-state donor fluorophore to the ground-state acceptor fluorophore (BODIPY skeleton). Thus, this work demonstrates that the PET/FRET-based fluorescent dye composed of the donor fluorophore possessing PET characteristics and the acceptor fluorophore in FRET process can act as a fluorescent sensor with large SS for the detection of a trace amount of water in solvents.

## Experimental

### General

Melting points were measured with a Yanaco micro melting point apparatus MP model. IR spectra were recorded on a PerkinElmer Spectrum One FT-IR spectrometer using ATR method. ^1^H and ^13^C NMR spectra were recorded on a Varian-400 (400 MHz) or a Varian-500 (500 MHz) FT NMR spectrometer. High-resolution mass spectral data by ESI and GC-FI were acquired on a Thermo Fisher Scientific LTQ Orbitrap XL and JEOL JMS-T100 GCV 4G, respectively. Photoabsorption spectra were observed with a SHIMADZU UV-3150 spectrophotometer. Fluorescence spectra were measured with a Hitachi F-4500 spectrophotometer. The fluorescence quantum yields were determined by a HORIBA FluoroMax-4 spectrofluorometer by using a calibrated integrating sphere system. The addition of water to acetonitrile solutions containing DJ-1 was made by weight percent (wt%). The determination of water in acetonitrile was done with a MKC-610 and MKA-610 Karl Fischer moisture titrator (Kyoto Electronics manufacturing Co., Ltd.) based on Karl Fischer coulometric titration for below 1.0 wt% and volumetric titration for 1.0–40 wt%, respectively.

### Synthesis

#### 2-(4-Bromo-3-methylphenyl)-4,4,5,5-tetramethyl-1,3,2-dioxaborolane (1)

To a diethylether solution (350 mL) of 1-bromo-4-iodo-2-methylbenzene (1.26 g, 42.4 mmol) at −78 °C under a nitrogen atmosphere was added dropwise 2.76 M hexane solution of *n*-BuLi (15.9 mL, 44.0 mmol). After stirring for 1 h at −78 °C, 2-isopropoxy-4,4,5,5-tetramethyl-1,3,2-dioxaborolane (8.84 g, 47.5 mmol) was added, and then, the reaction mixture was further stirred for 5 h. The mixture was quenched with 47% BF_3_–OEt_2_ (5.60 mL, 44.0 mmol), and then, the mixture was stirred and allowed to warm to room temperature. After stirring for 12 h at room temperature, the water was added to the reaction mixture, and then, the solution was extracted with diethylether. The diethylether extract was dried over MgSO_4_, filtrated, and evaporated under reduced pressure. The residue was chromatographed on silica gel (dichloromethane : hexane = 1 : 1 as eluent) to give 1 (10.0 g, 80% yield) as a white solid; mp 55–56 °C; FT-IR (ATR): *ν̃* = 2976, 1593, 1350, 1140, 1096 cm^−1^; ^1^H NMR (400 MHz, CDCl_3_): *δ* = 1.34 (s, 12H), 2.40 (s, 3H), 7.45 (dd, *J* = 1.1 and 7.9 Hz, 1H), 7.53 (d, *J* = 7.9 Hz, 1H), 7.66 (s, 1H) ppm; ^13^C NMR (125 MHz, CDCl_3_) *δ* = 22.78, 24.98, 84.10, 128.87, 132.00, 133.65, 137.17, 137.33 ppm (one aromatic carbon signal was not observed owing to overlapping resonances); HRMS (GC-FI): *m*/*z* (%): [M^+^˙] calcd for C_13_H_18_BBrO_2_, 296.05832; found 296.05863.

#### 2-(4-Bromo-3-(bromomethyl)phenyl)-4,4,5,5-tetramethyl-1,3,2-dioxaborolane (2)

A solution of 1 (11.4 g, 38.5 mmol), 1,3-dibromo-5,5-dimethylhydantoin (6.60 g, 23.1 mmol), and ZrCl_4_ (4.48 g, 19.2 mmol) in dichloromethane (400 mL) was stirred for 24 h at room temperature under ambient light. The water was added to the reaction mixture, and then, the solution was extracted with dichloromethane. The dichloromethane extract was dried over MgSO_4_, filtrated, and evaporated under reduced pressure. The crude product as a brown solid was recrystallized form *n*-hexane to give 2 (7.02 g, 50% yield) as a white solid; mp 100–102 °C; FT-IR (ATR): *ν̃* = 2976, 1593, 1350, 1138, 1092 cm^−1^; ^1^H NMR (500 MHz, CDCl_3_): *δ* = 1.34 (s, 12H), 4.61 (s, 2H), 7.56–7.60 (m, 2H), 7.86 (s, 1H) ppm; ^13^C NMR (125 MHz, CDCl_3_): *δ* = 24.99, 33.50, 84.38, 128.32, 133.04, 136.40, 136.58, 137.62, 137.70 ppm; HRMS (GC-FI): *m*/*z* (%): [M^+^˙] calcd for C_13_H_17_BBr_2_O_2_, 373.96884; found 373.96941.

#### 1-(Anthracen-9-yl)-*N*-(2-bromo-5-(4,4,5,5-tetramethyl-1,3,2-dioxaborolan-2-yl)benzyl)-*N*-methylmethanamine (3)

A solution of 2 (4.35 g, 11.6 mmol), 9-(methylaminomethyl)anthracene (2.56 g, 11.6 mmol), *N*,*N*-diisopropylethylamine (8.10 mL, 46.3 mmol), and acetonitrile (450 mL) was refluxed for 2 h under a nitrogen atmosphere. After concentrating under reduced pressure, the resulting residue was dissolved in dichloromethane and washed with water. The dichloromethane extract was dried over MgSO_4_, filtrated, and evaporated under reduced pressure. The resulting residue as a yellow solid was washed by hexane to give 3 (5.50 g, 92% yield) as a light-yellow solid; mp 149–150 °C; FT-IR (ATR): *ν̃* = 2973, 1594, 1349, 1140, 1095 cm^−1^; ^1^H NMR (400 MHz, CDCl_3_) *δ* = 1.34 (s, 12H), 2.22 (s, 3H), 3.86 (s, 2H), 4.51 (s, 2H), 7.42–7.50 (m, 4H), 7.53 (dd, *J* = 1.6 and 8.0 Hz, 2H), 7.58 (d, *J* = 7.9 Hz), 7.89 (d, *J* = 1.6 Hz), 7.98 (d, *J* = 8.1 Hz, 2H), 8.39 (s, 1H), 8.46 (d, *J* = 8.9 Hz, 2H) ppm; ^13^C NMR (125 MHz, CDCl_3_): *δ* = 25.02, 42.20, 53.24, 62.21, 84.12, 124.90, 125.38, 125.61, 127.54, 127.58, 129.02, 129.22, 130.33, 131.52, 131.53, 132.56, 135.06, 137.60, 138.56 ppm; HRMS (ESI): *m*/*z* (%): [M + H^+^] calcd for C_29_H_32_O_2_NBBr, 516.17040; found 516.17090.

#### 1-(Anthracen-9-yl)-*N*-((4-bromo-4′-(5,5-difluoro-1,3,7,9-tetramethyl-5*H*-4λ^4^,5λ^4^-dipyrrolo[1,2-*c*:2′,1′-*f*][1,3,2]diazaborinin-10-yl)-[1,1′-biphenyl]-3-yl)methyl)-*N*-methylmethanamine (4)

A solution of 3 (0.18 g, 0.35 mmol), [1-[(3,5-dimethyl-1*H*-pyrrol-2-yl)(3,5-dimethyl-2*H*-pyrrol-2-ylidene)methyl]-4-iodobenzene](difluoroborane) (0.16 g, 0.35 mmol), PdCl_2_(dppf)·CH_2_Cl_2_ (0.029 g, 0.035 mmol), and K_2_CO_3_ (0.29 g, 2.1 mmol) in DMF (22 mL) was stirred for 24 h at 80 °C under a nitrogen atmosphere. After concentrating under reduced pressure, the resulting residue was dissolved in dichloromethane and washed with water. The dichloromethane extract was dried over MgSO_4_, filtrated, and evaporated under reduced pressure. The residue was chromatographed on silica gel (dichloromethane : hexane = 2 : 1 as eluent) to give 4 (0.14 g, 56% yield) as an orange solid; mp 249–250 °C; FT-IR (ATR): *ν̃* = 2925, 1540, 1508, 1302, 1188, 1156, 975 cm^−1^; ^1^H NMR (400 MHz, CDCl_3_): *δ* = 1.44 (s, 6H), 2.39 (s, 3H), 2.56 (s, 6H), 3.84 (s, 2H), 4.62 (s, 2H), 6.01 (s, 2H), 7.42–7.50 (m, 4H), 7.31 (d, *J* = 8.2 Hz, 2H), 7.36 (dd, *J* = 2.3 and 8.2 Hz, 1H), 7.42–7.48 (m, 4H), 7.54–7.58 (m, 3H), 7.63 (d, *J* = 2.3 Hz, 1H), 7.99 (dd, *J* = 2.4 and 7.1 Hz, 2H), 8.41 (s, 1H), 8.52 (d, *J* = 7.7 Hz, 2H) ppm; ^13^C NMR (125 MHz, CDCl_3_): *δ* = 14.76, 14.80, 42.76, 53.60, 60.63, 121.39, 121.48, 124.25, 124.97, 125.25, 125.74, 126.80, 127.46, 127.76, 127.80, 128.61, 128.68, 129.20, 130.03, 130.08, 130.32, 131.48, 131.60, 133.13, 134.31, 138.89, 139.45, 140.44, 141.51, 143.24, 155.72 ppm; HRMS (ESI): *m*/*z* (%): [M + H^+^] calcd for C_42_H_38_N_3_BBrF_2_, 712.23047; found 712.23163.

#### 1-(Anthracen-9-yl)-*N*-((4′-(5,5-difluoro-1,3,7,9-tetramethyl-5*H*-4λ^4^,5λ^4^-dipyrrolo[1,2-*c*:2′,1′-*f*][1,3,2]diazaborinin-10-yl)-4-(4,4,5,5-tetramethyl-1,3,2-dioxaborolan-2-yl)-[1,1′-biphenyl]-3-yl)methyl)-*N*-methylmethanamine (DJ-1)

A solution of 4 (0.28 g, 0.40 mmol), bis(pinacolato)diboron (0.16 g, 0.63 mmol), PdCl_2_(dppf)·CH_2_Cl_2_ (0.034 g, 0.042 mmol), and KOAc (0.12 g, 1.3 mmol) in 1,4-dioxane (10 mL) was refluxed for 12 h under a nitrogen atmosphere. After concentrating under reduced pressure, the resulting residue was dissolved in dichloromethane, and washed with water. The dichloromethane extract was dried over MgSO_4_, filtrated, and evaporated under reduced pressure. The residue was dissolved in toluene, and HPLC was performed to give DJ-1 (0.1 g, 33% yield) as an orange solid; mp 274–276 °C; FT-IR (ATR): *ν̃* = 2976, 1605, 1542, 1509, 1344, 1305, 1189, 1157, 1072, 975 cm^−1^; ^1^H NMR (400 MHz, CDCl_3_): *δ* = 1.33 (s, 12H), 1.45 (s, 6H), 2.31 (s, 3H), 2.57 (s, 6H), 4.05 (s, 2H), 4.45 (s, 2H), 6.00 (s, 2H), 7.33 (d, *J* = 8.4 Hz, 2H), 7.40–7.42 (m, 4H), 7.59 (dd, *J* = 2.0 and 7.8 Hz, 1H), 7.70–7.72 (m, 3H), 7.92 (d, *J* = 7.7 Hz, 1H), 7.95–7.97 (m, 2H), 8.37–8.39 (m, 3H) ppm; ^13^C NMR (125 MHz, CDCl_3_): *δ* = 14.75, 14.79, 25.10, 43.00, 52.41, 61.65, 83.75, 121.37, 124.82, 124.85, 125.50, 125.58, 127.43, 127.78, 128.52, 129.00, 130.83, 131.52, 131.61, 134.19, 136.23, 141.47, 141.59, 143.34, 146.59, 155.61 ppm (four aromatic carbon signals were not observed owing to overlapping resonances); HRMS (ESI): *m*/*z* (%): [M + H^+^] calcd for C_48_H_50_O_2_N_3_B_2_F_2_, 760.40517; found 760.40631.

## Conflicts of interest

There are no conflicts to declare.

## Supplementary Material

RA-009-C9RA02686J-s001
