# Supplementary material for: Fluorescent sensor for water based on photo-induced electron transfer and Förster resonance energy transfer: anthracene-(aminomethyl)phenylboronic acid ester-BODIPY structure
Source: RSC Adv. 2019 May 16;9(27):15335–40. doi: 10.1039/c9ra02686j (PMC9064233; doi:10.1039/c9ra02686j)
Supplement: RA-009-C9RA02686J-s001 [file RA-009-C9RA02686J-s001.pdf]

Electronic Supplementary Material (ESI) for RSC Advances.
This journal is © The Royal Society of Chemistry 2019

Supplementary Information

Fluorescent sensor for water based on photo-induced electron transfer and Förster resonance energy transfer: anthracene-(aminomethyl)phenylboronic acid ester-BODIPY structure

Daisuke Jinbo, Keiichi Imato and Yousuke Ooyama *

Department of Applied Chemistry, Graduate School of Engineering, Hiroshima University, Higashi-Hiroshima, 739-8527, Japan.

Fax: +81 824 24 5494; Tel: +81 824 24 7689; E-mail:yooyama@hiroshima-u.ac.jp

Synthesis of 10-([1,1'-Biphenyl]-4-yl)-5,5-difluoro-1,3,7,9-tetramethyl-5*H*-4λ^4,5λ^4-dipyrrolo[1,2-*c*:2',1'-*f*][1,3,2]diazaborinine (B-1)

A solution of 5,5-difluoro-10-(4-iodophenyl)-1,3,7,9-tetramethyl-5*H*-4λ^4,5λ^4-dipyrrolo[1,2-*c*:2',1'-*f*][1,3,2]diazaborinine (0.050 g, 0.11 mmol), phenylboronic acid (0.016 g, 0.13 mmol), $PdCl_2(PPh_3)_2$ (0.017 g, 0.024 mmol), and K_2CO_3 (0.061 g, 0.44 mmol) in 1,4-dioxane (9 mL) and water (0.44 mL) was refluxed for 12 h under a nitrogen atmosphere. After concentrating under reduced pressure, the resulting residue was dissolved in dichloromethane, and washed with water. The dichloromethane extract was dried over Na_2SO_4, filtrated, and evaporated under reduced pressure. The residue was chromatographed on silica gel (dichloromethane-hexane = 2 : 1 as eluent) to give **B-1** (0.027 g, 61 % yield) as an orange solid; ^{1}H NMR (400 MHz, $CDCl_3$): δ = 1.45 (s, 6H), 2.57 (s, 6H), 5.99 (s, 2H), 7.35 (d, J = 8.3 Hz, 2H), 7.40 (t, 1H), 7.40 (t, 2H), 7.69 (d, J = 7.2 Hz, 2H), 7.75 (d, J = 8.3 Hz, 2H) ppm; HRMS (ESI): *m*/*z* (%):[M+Na$^+$] Calcd for $C_{25}H_{23}N_2BF_2Na$, 423.18146; found423.18143.

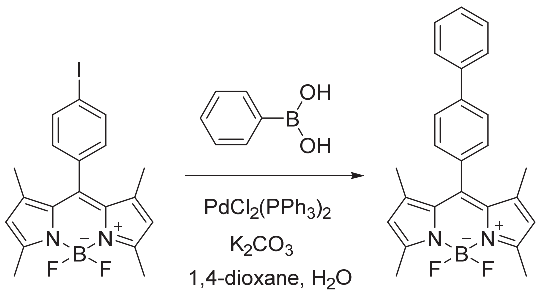

B-1

Scheme S1 Synthesis of **B-1**

(a)

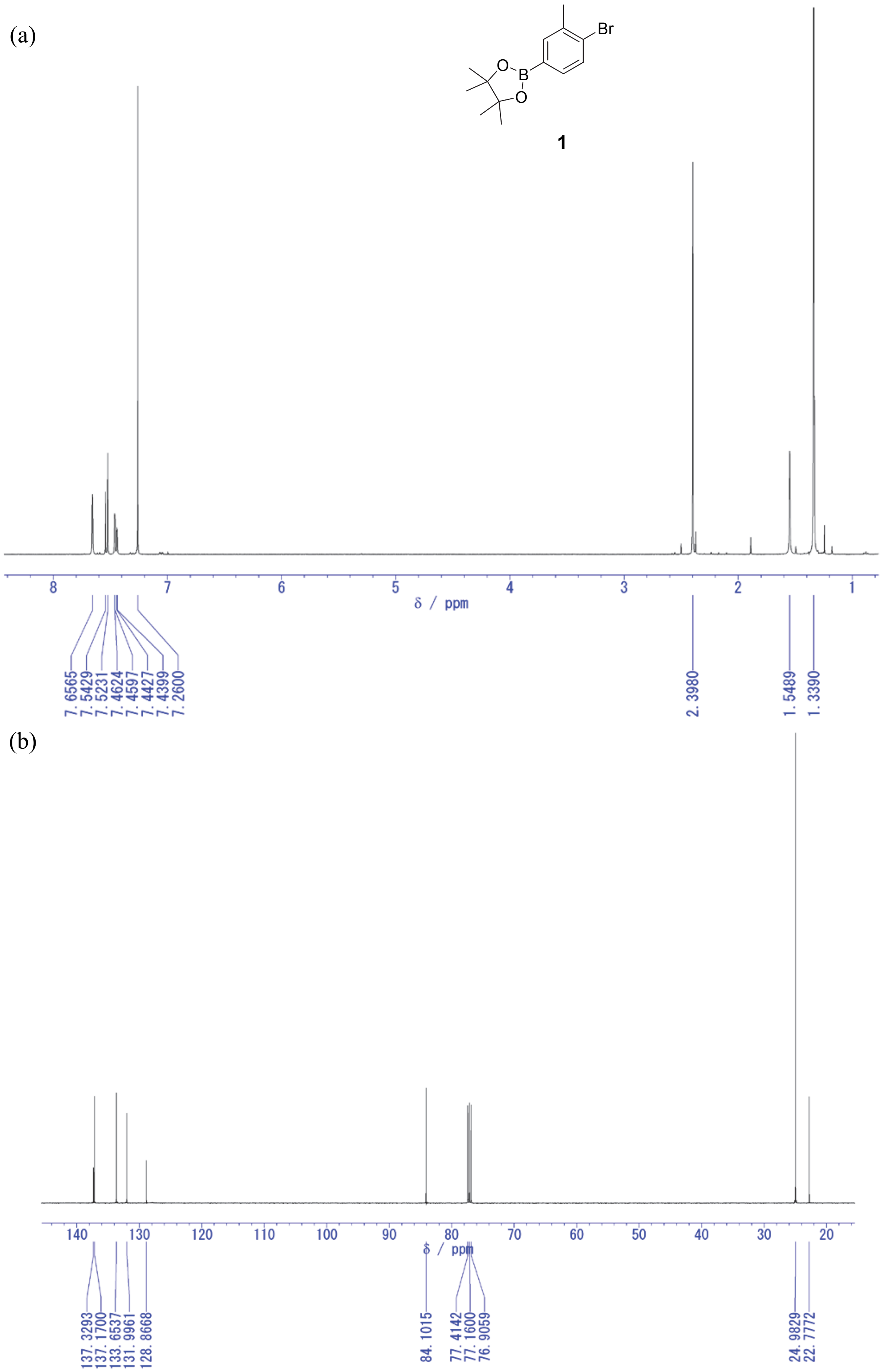

Fig. S1 (a) ^{1}H HMR (400 MHz) and (b) ^{13}C HMR (125 MHz) spectra of **1** in $CDCl_3$.

(a)

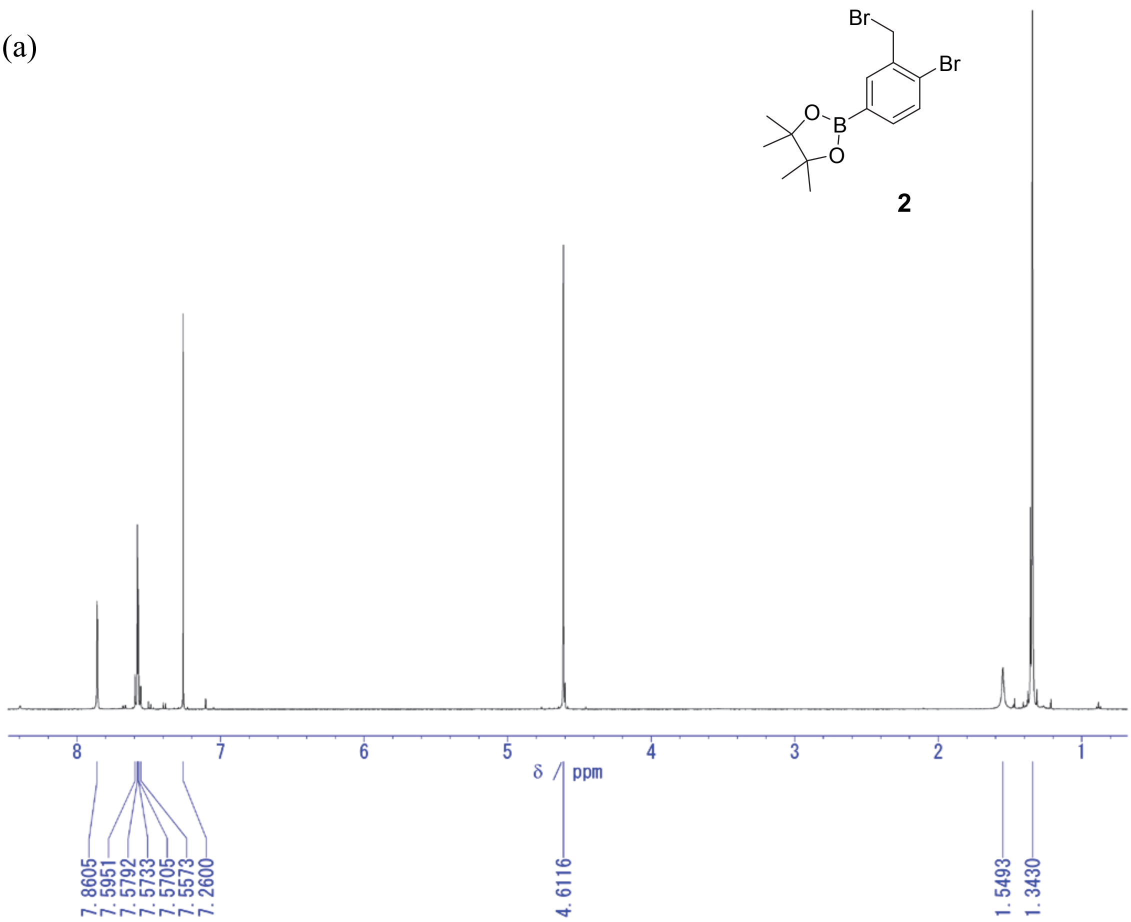

(b)

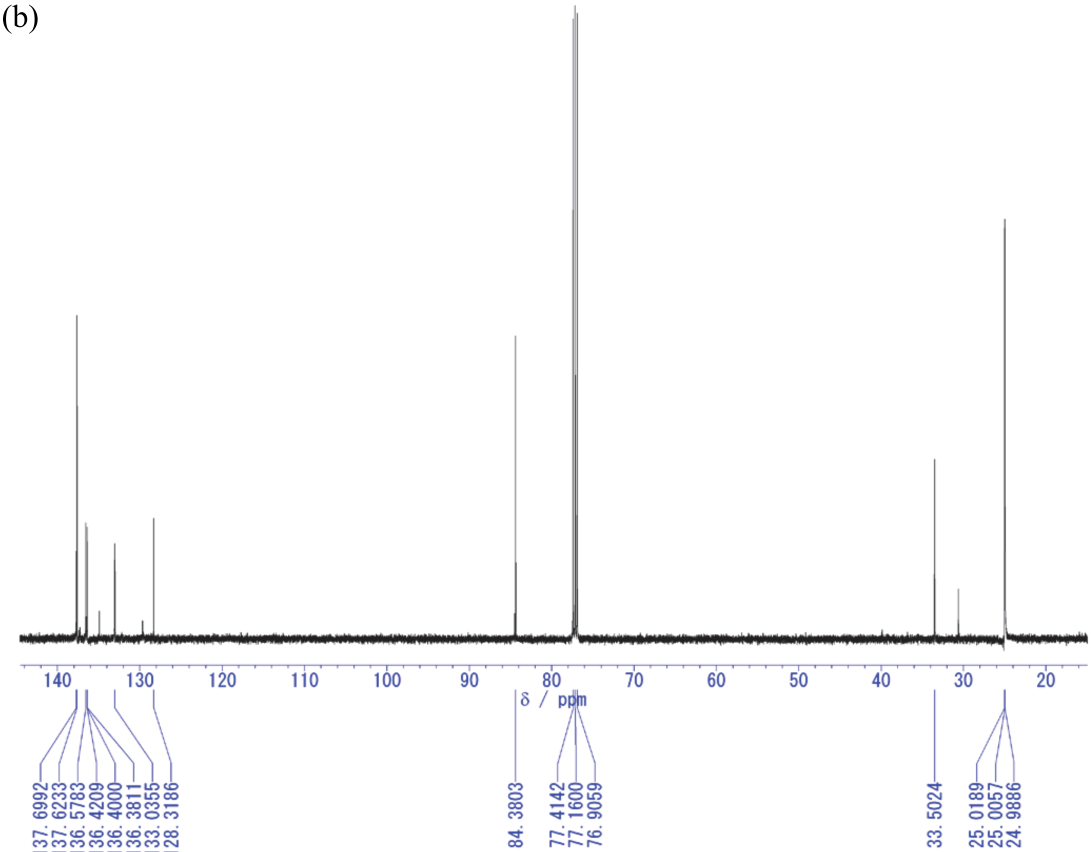

Fig. S2 (a) ^{1}H HMR (500 MHz) and (b) ^{13}C HMR (125 MHz) spectra of **2** in $CDCl_3$.

(a)

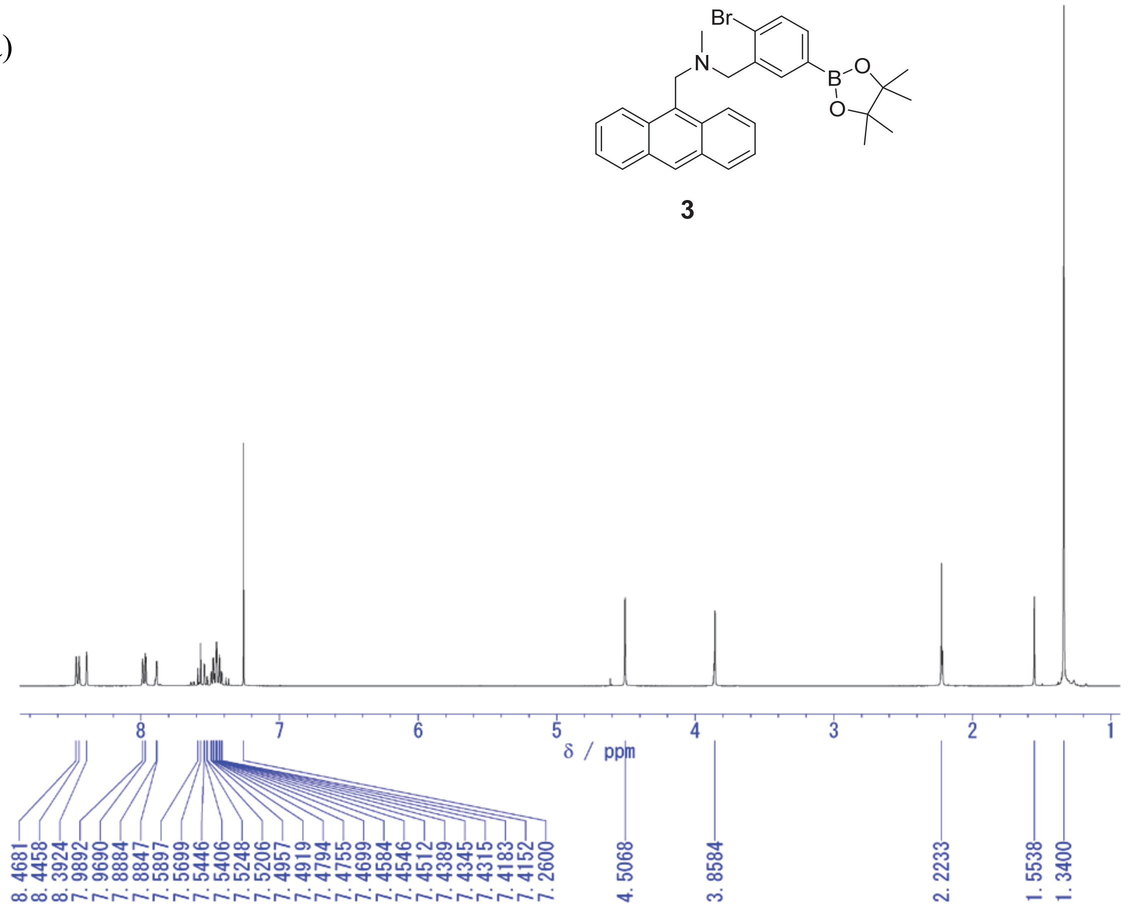

(b)

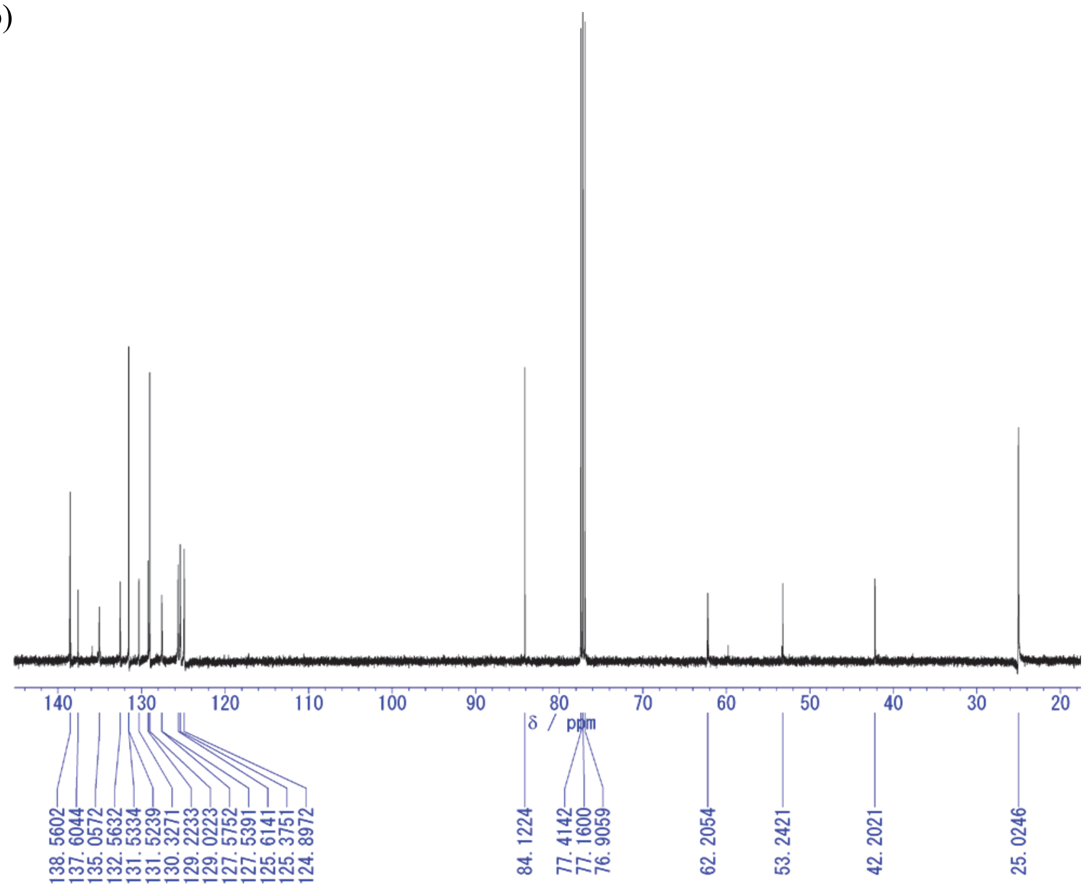

Fig. S3 (a) ^{1}H HMR (400 MHz) and (b) ^{13}C HMR (125 MHz) spectra of **3** in $CDCl_3$.

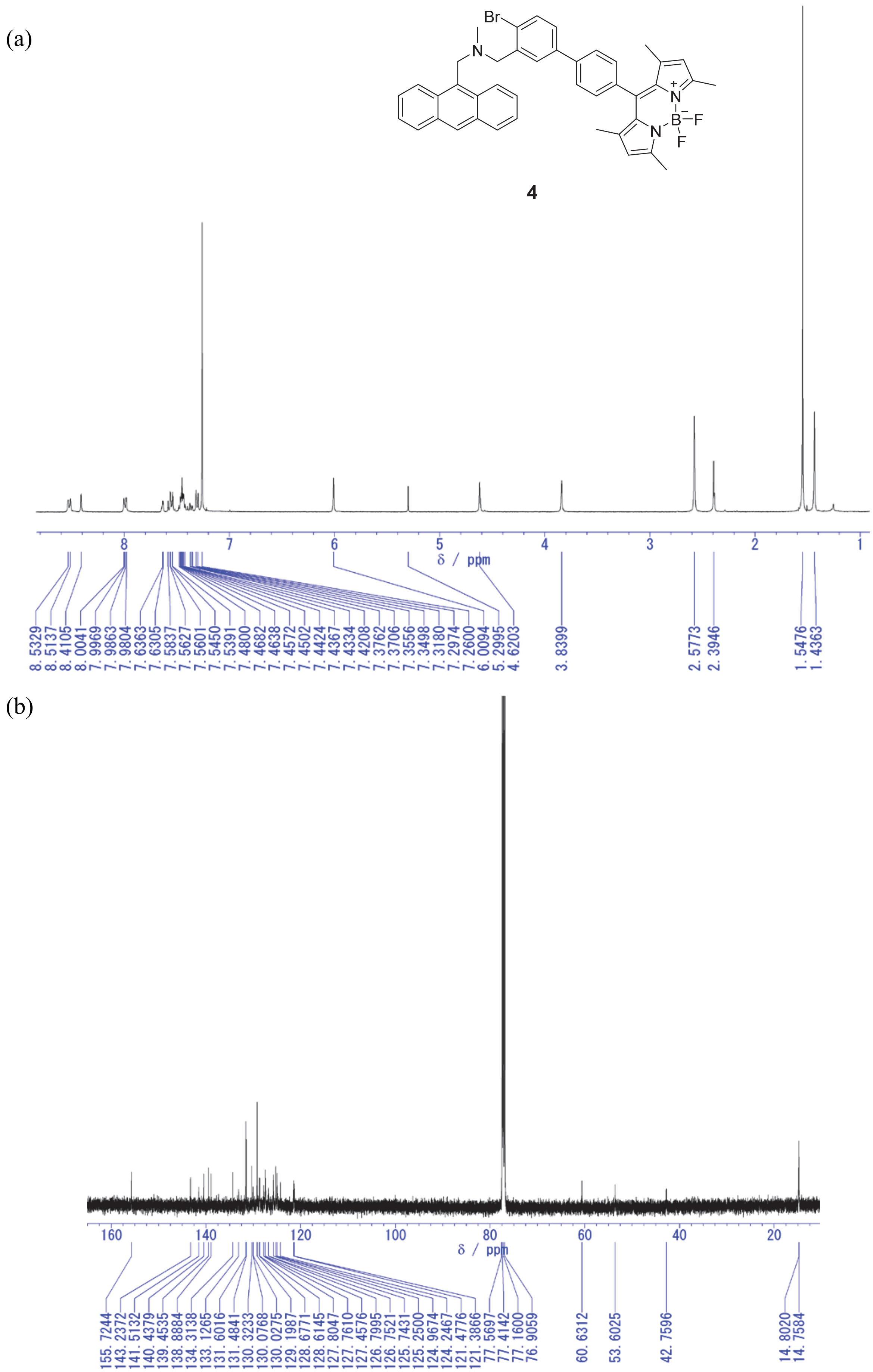

Fig. S4 (a) ^{1}H HMR (400 MHz) and (b) ^{13}C HMR (125 MHz) spectra of **4** in $CDCl_3$.

(a)

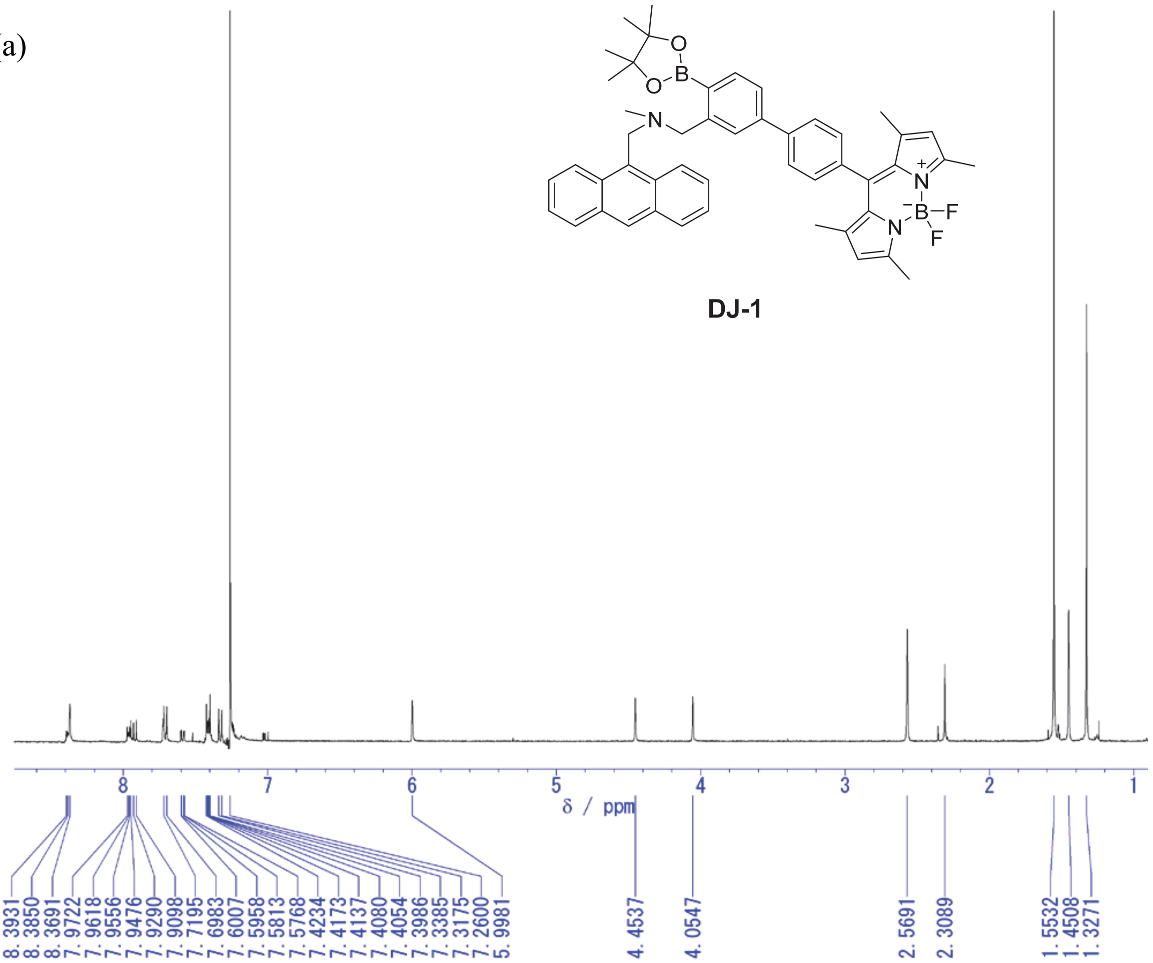

(b)

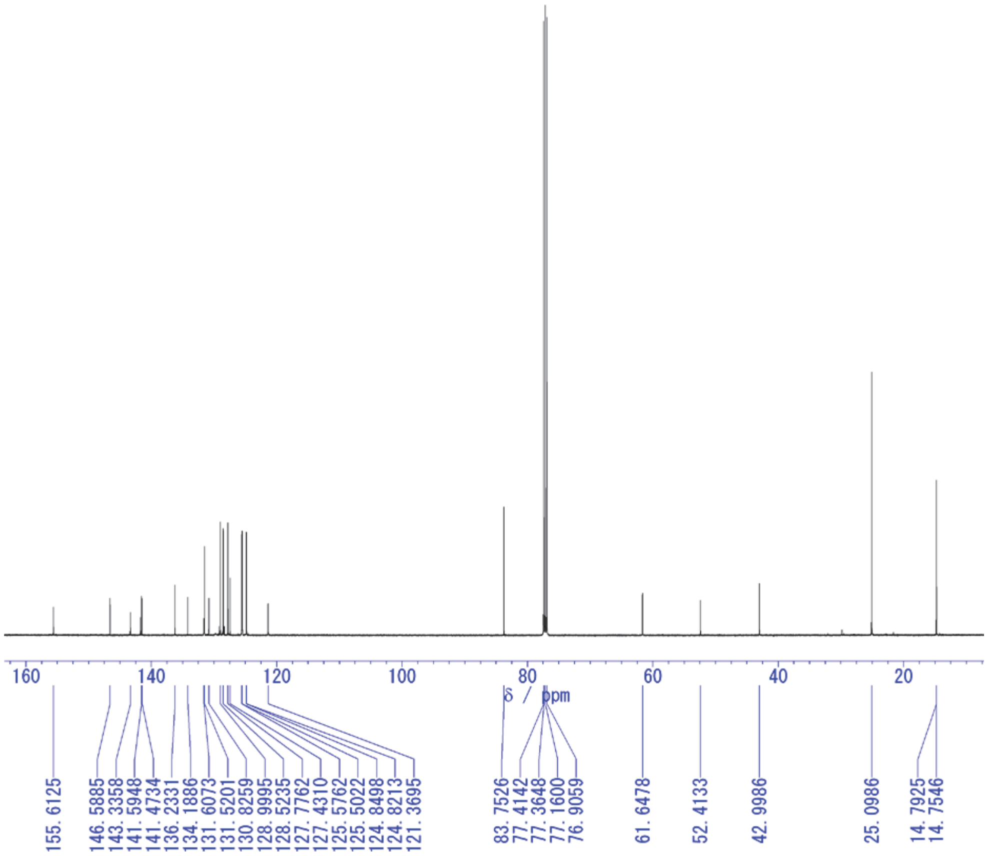

Fig. S5 (a) ^{1}H HMR (400 MHz) and (b) ^{13}C HMR (125 MHz) spectra of **DJ-1** in $CDCl_3$.

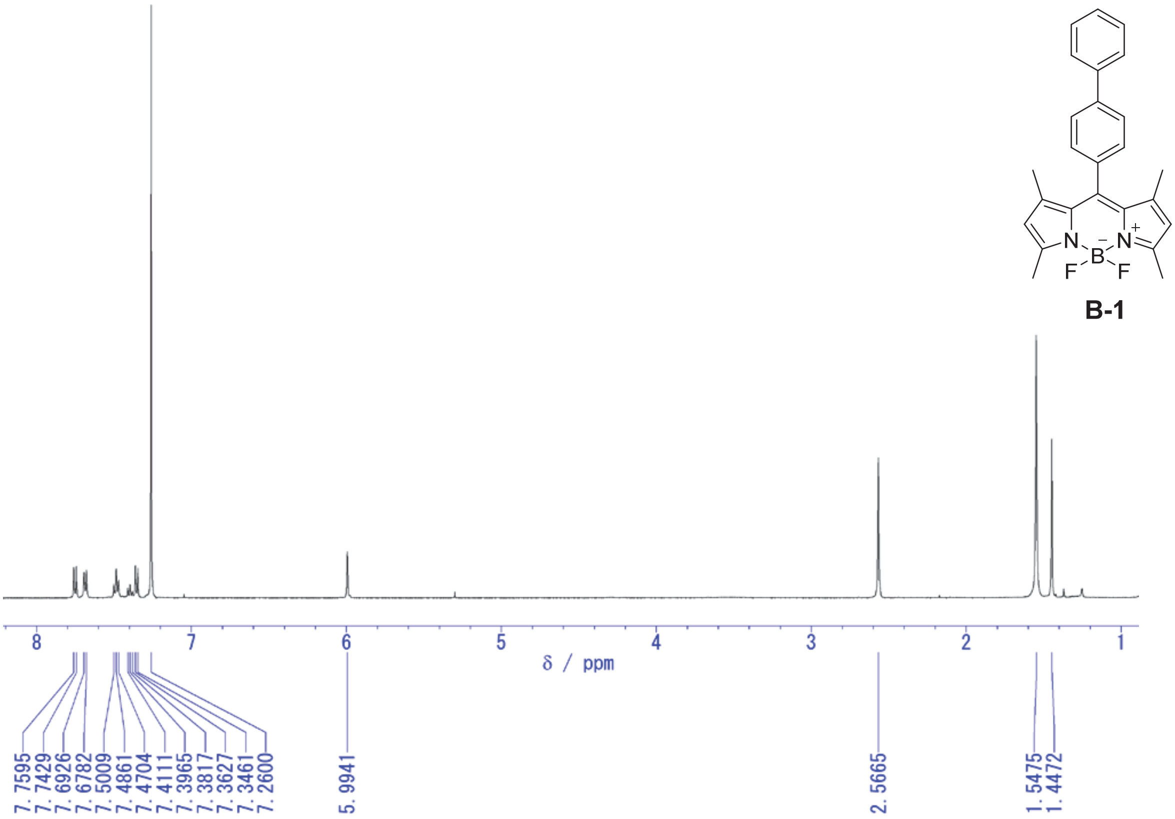

Fig. S6 ^{1}H HMR (400 MHz) spectrum of **B-1** in $CDCl_3$.

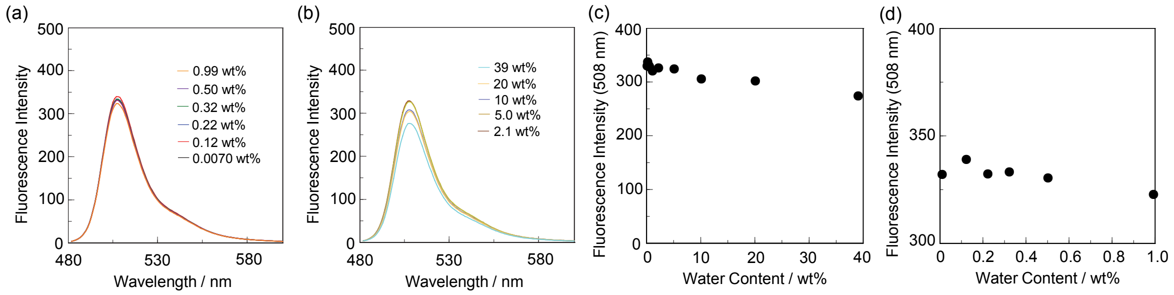

Fig. S7 Fluorescence spectra (λ^{ex} = 472 nm) of **DJ-1** (c = 4.0×10^{-6} M) in acetonitrile containing (a) 0.007–0.99 wt% and (b) 2.1–39 wt% of water. Fluorescence peak intensity at 508 nm of **DJ-1** (λ^{ex} = 472 nm) as a function of water content (c) below 40 wt% and (d) 1.0 wt% in acetonitrile.

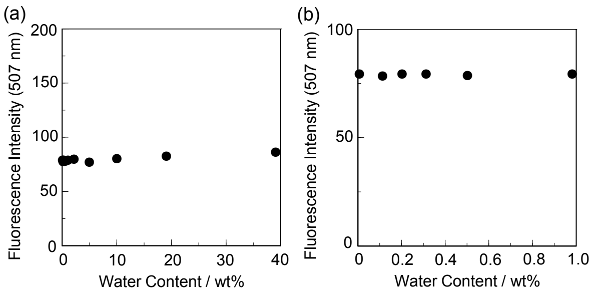

Fig. S8 Fluorescence peak intensity at 507 nm of **B-1** (λ^{ex} = 367 nm) as a function of water content (a) below 40 wt% and (b) 1.0 wt% in acetonitrile.
